# Palmitoylation: a protein *S*-acylation with implications for breast cancer

**DOI:** 10.1038/npjbcancer.2016.28

**Published:** 2016-10-19

**Authors:** Alison M Anderson, Mark A Ragan

**Affiliations:** 1 Institute for Molecular Bioscience, The University of Queensland, Brisbane, QLD, Australia

## Abstract

Protein *S*-acylation is a reversible post-translational lipid modification that involves linkage of a fatty acid chain predominantly to a cysteine amino acid via a thioester bond. The fatty acid molecule is primarily palmitate, thus the term ‘palmitoylation’ is more commonly used. Palmitoylation has been found to modulate all stages of protein function including maturational processing, trafficking, membrane anchoring, signaling range and efficacy, and degradation. In breast cancer, palmitoylation has been shown to control the function of commonly dysregulated genes including estrogen receptors, the epidermal growth factor (EGF) family of receptors, and cancer stem cell markers. Importantly, palmitoylation is a critical factor controlling the formation of complexes at the plasma membrane involving tetraspanins, integrins, and gene products that are key to cell–cell communication. During metastasis, cancer cells enhance their metastatic capacity by interacting with stroma and immune cells. Although aberrant palmitoylation could contribute to tumor initiation and growth, its potential role in these cell–cell interactions is of particular interest, as it may provide mechanistic insight into metastasis, including cancer cell-driven immune modulation. Compelling evidence for a role for aberrant palmitoylation in breast cancer remains to be established. To this end, in this review we summarize emerging evidence and highlight pertinent knowledge gaps, suggesting directions for future research.

## Introduction

Breast cancer is a heterogeneous disease commonly categorized into three distinct therapeutic groups based on the expression of estrogen receptor alpha (ER/ESR1), progesterone receptor (PR) and the epidermal growth factor receptor HER2/ERBB2.^1^ Metastasis to other organs, not cancer of the breast itself, is the major cause of mortality in patients with malignancies.^2^ Metastatic disease is generally understood as a linear succession of events commonly referred to as the metastatic cascade, in which cells from a primary tumor invade the local extracellular matrix and stroma, intravasate into the blood and/or lymphatic vessels, and subsequently arrest and engraft at distal sites.^3^ Cancer stem cells (CSCs) with enhanced capacity to disseminate from the primary tumor, and stemness and self-renewal properties that enable tumor-initiating ability at distal sites, are considered to be important drivers of metastasis.^4^ The activity of CSCs is also coupled with compromise or dysfunction of the immune system.^4,5^

Metastatic potential differs across therapeutic groups, and the location and receptor status of tumor(s) at the first site of metastasis have important consequences on the spatiotemporal progression of the disease.^6^ How dysregulation of the classical receptors (ESR1 and ERBB2) relates to mechanisms driving metastasis including CSC activity and immune modulation remains to be fully understood. Aberrant palmitoylation is potentially a unifying factor. Importantly, the molecular components and dynamics of the palmitome might harbor novel therapeutic targets. For instance, treatments directed at the post-translational level could mitigate dysregulation of the primary biomarkers, providing an alternative for patients who are refractory to drugs that target overexpressed receptors, or present with receptor-negative tumors. Compelling evidence for a role for aberrant palmitoylation in breast cancer remains to be established. To this end, we summarize evidence that links palmitoylation to critical hallmarks of breast cancer, and highlight knowledge gaps, suggesting directions for future research. We begin with a brief introduction to palmitoylation.

### *S*-acylation/palmitoylation

Acylation is a protein modification in which an acyl group is covalently linked to a cysteine, serine, or threonine residue.^7^ Protein *S*-acylation is a subtype that involves linkage of the fatty acid chain predominantly to a cysteine amino acid in a thioester bond. This process was first described in viral glycoprotein maturation^8^ and subsequently found to be ubiquitous and highly conserved from yeast to human.^7^ Because the fatty acid molecule is primarily palmitate the term ‘palmitoylation’ is more commonly used, but other saturated (e.g., myristic and stearic) and unsaturated (e.g., oleic and arachidonic) fatty acids also form modifications. Acylation can also occur via an ester (*O*-acylation) or amide (*N*-acylation) linkage.^9^

Palmitoylation is enzymatically reversible, with variable cycling rates observed across substrates within and across proteins.^10^ These dynamic cycles have been shown to mediate all stages of protein function including maturational processing, trafficking, membrane anchoring, signaling range and efficacy, and degradation.^9^ Consequently, palmitoylation events are critical for normal cellular function and impact on diverse biological processes ranging from synaptic plasticity^11^ to immune regulation.^12^ There is evidence for non-enzymatic palmitoylation, but enzymatic mechanisms predominate. Palmitoylation and depalmitoylation processes are catalysed by protein acyltransferases (PATs) and acyl-protein thioesterases (APTs), respectively.^13,14^

### Protein acyltransferases

**Figure 1:**
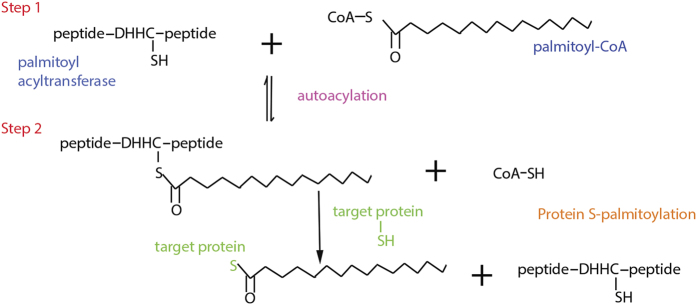
Two steps of palmitoylation (modified from William Christie, http://www.lipidhome.co.uk/lipids/simple/protlip/index.htm, with permission).

PATs contain a DHHC cysteine-rich domain and were first described in yeast.^15^ Subsequently, 24 PATs were identified in human and designated ZDHHC1 to 24 (without ZDHHC10 and including ZDHHC11B).^16^ Palmitoylation proceeds via a two-step catalytic mechanism. In the first step, the cysteine residue in the DHHC motif is acylated with palmitate from palmitoyl-CoA (this step is often referred to as auto-palmitoylation, as APTs incubated with palmitoyl-CoA are spontaneously palmitoylated). In the second step, the palmitoyl moiety is transferred to the thiol of a cysteine in a protein substrate (Figure 1). In the absence of a protein substrate, hydrolysis of the ester bond results in the release of palmitate and the APT is free to repeat the process. Substrate specificity has been shown to vary across PAT family members and is yet to be fully characterized.^17^

Many PATs have been reported to act as tumor suppressors, oncogenes, or both.^18^ Indeed, following observations from murine cell studies ZDHHC2 was originally named ream*,* for reduced expression associated with metastasis*.*^19^ These authors noted that the gene maps to a region that is frequently lost in many cancers including breast cancer.^19^ PATs specifically implicated in breast cancer include ZDHHC7 and ZDHHC21, which are responsible for the palmitoylation of the sex steroid receptors estrogen (ER), progesterone (PR), and androgen (AR);^20^ ZDHHC17 and ZDHHC20, upregulated in breast cancer cells;^21,22^ and ZDHHC9, which induces hyperproliferation when overexpressed in the premalignant breast cancer cell line MCF10AT.^23^

### Acyl-protein thioesterases

Palmitoyl protein thioesterase (PPT1), a liposomal resident, was the first enzyme shown to catalyse the removal of fatty acids from proteins. Two cytoplasmic APT enzymes were subsequently identified that also act as lysophospholipases and are thus named LYPLA1/APT1 and LYPLA2/APT2.^24^ Some evidence suggests that PPT1 may also be active in the cytoplasm.^25^ Hsu *et al.*^26^ reported that the retinoid-inducible tumor suppressor RARRES3/HRASLS4 acts as an APT to regulate Wnt proteins in breast cancer cells.^26^ RARRES3 was previously reported to display phospholipase PLA1/2 activities^27^ and concomitant lysophosphatidylcholine (LPC) *O*-acylation and phosphatidylethanolamine *N*-acylation.^28^ The capacity for APTs to confer multiple lipid modifications indicates that the enzymatic profile of many lipid-modifying enzymes may remain to be fully characterized. Furthermore, the interdependence and functional implications of simultaneous lipid-based events are yet to be fully explored.

As with PATs, the involvement of APTs is increasingly reported in the cancer literature. PPT1 has been associated with tumor progression and metastasis in colorectal cancer,^29^ is a potential biomarker for bladder cancer^30^ and is a direct target of the antineoplastic cyclic depsipeptide didemnin B.^31^ LYPLA1 is reported in depalmitoylation pathways in association with prostate cancer,^32^ endometrial cancer,^33^ and chronic lymphocytic leukemia.^34^ Interestingly, the Wnt family member Wnt5a promotes depalmitoylation of the cell adhesion molecules CD44 (a well-known CSC marker) and MCAM in melanoma cells via LYPLA1.^35^ This study found that three mutations in the ZDHHC20 PAT, observed in melanoma patients, increase the levels of palmitoylated enzyme as compared with wild type, and may therefore block capacity of the enzyme to enact the second step of palmitoylation catalysis (the transfer of palmitate from ZDHHC20 to the MCAM protein); and that depalmitoylation of MCAM alone is not necessarily sufficient to promote cell invasion, whereas overexpression of LYPLA1 is sufficient.

### Palmitoylation and its possible role in breast cancer metastasis

Many genes associated with metastasis are functionally controlled by palmitoylation; however, a specific role for aberrant palmitoylation in their dysregulation or pathological activity remains to be investigated. The most-direct evidence linking perturbation of palmitoylation cycling to metastatic disease involves two proteins that modulate the post-translational modification, ZDHHC2 and RARRES3. The latter displays capacity for depalmitoylation; it was identified in gene signatures specific to bone and lung metastasis, and in breast cancer cells has been shown to modulate Wnt protein palmitoylation.^26,36,37^ ZDHHC2 is located at chromosome 8p21.3–22, which is frequently deleted or affected by loss of heterozygosity in metastatic cancers.^18^ An association between chromosome arm 8p and progression of breast cancer has been known for two decades,^38,39^ but the relevance of ZDHHC2 loss is only beginning to be understood.

ZDHHC2 regulates tetraspanins CD9 and CD151.^40^ Tetraspanins are small membrane proteins that contribute to cell motility, adhesion, invasion, and immune-signaling mechanisms. They have been implicated in many cancers, and act as both metastasis suppressors and metastasis promoters.^40^ How they influence metastasis appears to be cancer type-specific and depends on the composition of tetraspanin complexes. Palmitoylation is an essential mechanism controlling the formation of the complexes, which involves interactions between the tetraspanins themselves and other transmembrane and cytosolic proteins, including proteins that are also functionally regulated by palmitoylation, and have independently been implicated in metastasis-promoting mechanisms. The latter include CD44v6,^41,42^ EGFR,^43,44^ and integrin alpha 6 beta 4/ITGA6.^45,46^ Thus, loss of ZDHHC2 or RARRES3 indirectly impacts on mechanisms associated with metastasis by altering the interaction dynamics of their target proteins, many of which are key modulators of cell function and operate at cellular membranes. Although aberrant palmitoylation can contribute to tumor initiation and growth, its potential role in these cell–cell interactions is of particular interest as it may provide new mechanistic insights into metastatic disease.

It will be important to determine the specific implications of palmitoylation perturbation on key breast cancer metastasis genes whose functions depend on palmitoylation in other cell types. For example, in 2007 Weinberg and colleagues showed how the tumor environment facilitates metastatic spread by eliciting reversible changes in the phenotype of cancer cells via CCL5-CCR5 chemokine signaling involving mesenchymal stem cells.^47^ CCR5 also contributes to breast cancer metastasis to bone by mediating the interaction between cancer cells and fibroblasts in the bone cavity.^48^ As with most chemokines, CCR5 undergoes palmitoylation at three cysteine residues within its C-terminal region. In immune cells, palmitoylation controls transport of the receptor to the cell surface and its interaction with signaling pathways. Eliminating palmitoylation reduces surface expression by trapping the receptor in organelles, where it is degraded.^49,50^ Better understanding of the role for palmitoylation in controlling surface-level expression of CCR5 in breast cancer cells could elucidate its role in mediating cancer cell interactions with other cell types that support the metastasis process.

Differential perturbation of palmitoylation might also provide mechanistic insight into breast cancer subtype-specific patterns of metastasis. Here again, palmitoylation-focussed investigations are required to build on current evidence arriving from disparate studies. For example MUC1, a mucin-like protein on the apical membrane of epithelia, is associated with metastasis.^51^ In hamster ovary cells palmitoylation plays a role in modulating MUC1 recycling from endosomes to the plasma membrane,^52^ and intracellular localization of MUC1 is altered in a breast cancer subtype-specific manner during tumorigenesis.^53^

## Palmitoylation and estrogen receptors

Estrogen receptor alpha (ESR1) is the major hormone receptor used to categorize patients with breast cancer, and most tumors are ESR1-positive.^54^ Expression of ESR1 in breast cancer tissue has been measured in patients for over 40 years and was initially determined using ligand-binding assays. Over time it has become increasingly clear that the function of ESR1 is highly nuanced and depends on the activity of many other molecules involved in estrogen signaling cascades,^55^ including other forms of estrogen receptors.^56^ The regulation of ESR1 continues to be elucidated^57^ in which the role of post-translational modifications is only beginning to be understood.^58^

**Figure 2:**
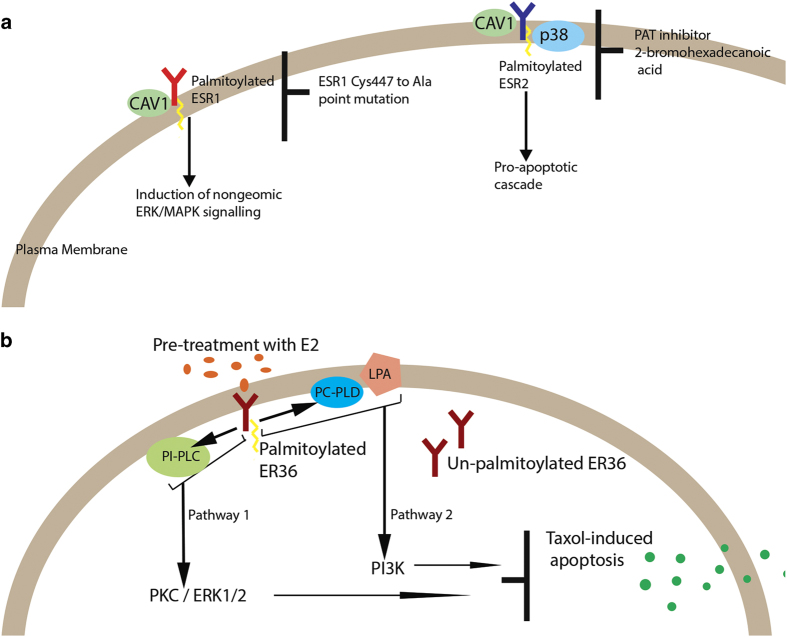
(**a**) The ability of ESR1 and ESR2 to associate with scaffolding and/or signaling proteins at the plasma membrane is principally due to palmitoylation. Mutation of ESR1 Cys447 to Ala prevents palmitoylation, plasma membrane localization, association with caveolin-1 (CAV1), and non-genomic activities.^56^ Palmitoylation of ESR2 was established in a human colon adenocarcinoma cell line, which contains only one ESR2 isoform.^61^ ESR2 association with caveolin-1 and p38 was prevented by pretreatment with the protein acyltransferases (PAT) inhibitor 2-bromohexadecanoic acid. (**b**) E2-induced membrane-associated ER36 mediates anti-apoptotic effect in triple-negative breast cancer cells. Palmitoylated ER36 translocates to the plasma membrane and enacts two independent pathways: PI3K signaling that requires interaction with phosphatidylcholine-specific phospholipase D (PC-PLD) and lysophosphatidic acid (LPA), and ERK1/2 signaling involving phosphatidylinositol-specific phospholipase C (PI-PLC) and protein kinase C (PKC).^62^

A role for palmitoylation in estrogen-receptor signaling was first identified in 2003 when plasma membrane recruitment of an alternatively spliced isoform of ESR1, designated ER46 (46 kDa), was found to be palmitoylation dependent.^59^ Around the same time the Marino laboratory showed that palmitoylation targets the canonical receptors ESR1 and ESR beta (ESR2) to plasma membrane caveolae thereby enabling their subsequent interaction with signal-transduction molecules (Figure 2a).^56,60^ This work provided several useful insights. The sex steroid 17 beta-estradiol (E2) reduces palmitoylation in a time- and dose-dependent manner.^56^ Palmitoylation cycling is more rapid for ESR1 than for ESR2, and depalmitoylation of these receptors mediates opposite functions on E2-exposed cancer cells: E2-induced depalmitoylation drives proliferation pathways (ERK/MAPK, PI3K/AKT, and PKC signaling associated with cell survival and cell cycle modulation, e.g., cyclin D1), whereas depalmitoylation of ESR2 drives proapoptotic activity (p38/MAPK signaling cascade involving caspase-3 activation and PARP cleavage).^61^

The Levin laboratory^20^ extended this work by identifying a palmitoylation motif within the ligand-binding (E) domain of all sex-steroid receptors. In addition, the binding of heat shock protein (hsp)27 to the palmitoylation motif of ESR1, PR, and androgen receptor (AR) was found to promote palmitoylation. In the case of ESR1, the authors noted that palmitoylation can take place in the cytosol and involves monomeric forms of the receptor, indicating that E2 might control receptor palmitoylation by inducing dimerization and thus limiting the amount of monomeric ESR1 that is available to undergo palmitoylation.

Interestingly, palmitoylation-dependent plasma membrane localization and activation of a second isoform of ESR1, designated ER36 (36 kDa), has been described in triple-negative breast cancer cells that are generally considered to be negative for all receptors (ESR1, ERBB2, and PR).^62^ E2-induced ER36 was found to mediate anti-apoptotic effects in cells treated with the chemotherapeutic taxol, through two signaling pathways (Figure 2b). Importantly, both estrogens and anti-estrogens, including the common therapeutic tamoxifen, stimulate cell growth via ER36 and the MAPK/ERK pathway, with anti-estrogens showing a stronger activation of MAP/ERK.^63^ These observations have implications for the treatment of patients resistant to tamoxifen.

### Knowledge gaps relating to estrogen receptor palmitoylation

These independent reports provide insight into the importance of palmitoylation-dependent regulation of estrogen receptor function, but many fundamental questions remain to be addressed. In addition to palmitoylation, ESR1 undergoes phosphorylation, acetylation, methylation, ubiquitination, and sumoylation.^64–69^ To what extent these modifications are interdependent is not clear. ESR2 has multiple isoforms for which a role for palmitoylation (and other post-translational modifications (PTMs)) is yet to be investigated. A better understanding of the post-translational landscape could explain why ESR1 and its alternative isoforms ER36 and ER46 share ligand- and DNA-binding domains but display different functions.^58^ Similarly, the mechanisms underlying the differential binding affinity of the receptor types for E2 and other estrogen-receptor agonists and antagonists remain elusive.^70^

A further gray area is the role of palmitoylation in mediating crosstalk among estrogen receptors, and also among these receptors and members of the EGF family of proteins that are also dysregulated during breast cancer. In ESR1-positive breast cancer cells, ESR1 and ER46 co-localize with EGFR and ERBB2 in lipid rafts,^71^ and in ESR1-negative cells a positive-feedback loop involving ER36 and EGFR promotes malignant growth.^72^ Differential perturbations of coactivity among the estrogen and EGF family receptors might contribute to the many different breast cancer subtypes described using whole-genome transcriptomic approaches, which often appear to be disconnected from the activity of the traditional clinical markers.^73^

**Figure 3:**
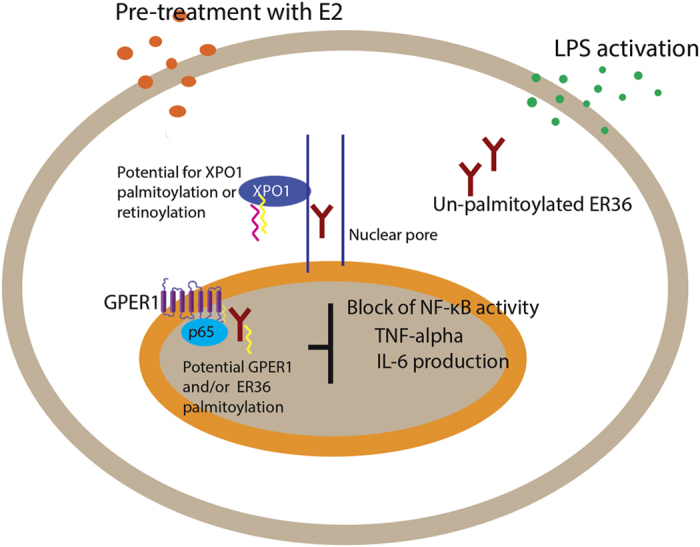
Anti-inflammatory action of E2 on human primary monocytes/macrophages is mediated in part through ER36 and GPER1. LPS-induced inflammatory response leads to the secretion of proinflammatory mediators including IL-6 and TNF-alpha mediated by NF-κB activity within the nucleus. Pretreatment with E2 blocks this action through a transiently localized ER36 and NF-κB p65 subunit. GPER1 physically interacts with ER36 and p65 acting as a co-regulator.^77^ Nucleocytoplasmic trafficking of ER36 requires exportins. Potential palmitoylation-dependent mechanisms include localization of ER36 at nuclear membrane (as reported in plasma membrane of breast cancer cells^62^), activity of the G-protein-coupled receptor GPER1, and the activity of XPO1 which may be palmitoylation dependent^133^ and/or retinoylation-dependent. IL, interleukin; LPS, lipopolysaccharide; TNF, tumor necrosis factor.^79^

Estrogen signaling has a complex and important role in innate and adaptive immune systems, the nuances of which are beyond the scope of this review.^74–76^ Of particular interest here is a study by Pelekanou *et al.*^77^ in monocytes and macrophages that identified a role for ER36 and GPER1. ER36 was shown to mediate E2-induced anti-inflammatory activity, and was transiently active within the nucleus where it physically interacted with GPER1.^77^ A role for palmitoylation was not investigated, but localization of ER36 to the nuclear membrane is palmitoylation dependent,^62^ and GPER1 is a G-protein-coupled receptor, most of which undergo palmitoylation.^78^ Given that nucleocytoplasmic trafficking of ER36 is dependent on exportins^77^ and the exportin (XPO1/CRM1) is palmitoylation-regulated and undergoes retinoylation as discussed later in this review,^79–81^ further post-translational mechanisms may be in play (Figure 3). However, this overall conjecture is likely oversimplified. Both membrane localization and function of ESR1, and ER36 inhibition of estrogen-dependent and estrogen-independent transactivation of ESR1 and ESR2, are palmitoylation dependent.^56,63^ Indeed in murine lymphocytes, fine-tuning of immune response involves a complex dynamic involving multiple forms of estrogen receptors.^82^ Pelekanou *et al.*^77^ evaluated all known E2-binding isoforms of ESR1, ESR2, GPER1 and AR in monocytes and macrophages, and found only GPER1 and ER36 to be expressed. However, a role for ESR1 in mediating E2-induced expression of inflammatory molecules in macrophages has been described.^83^ This contridiction may arise from differences in experimental conditions, or might reflect cellular environment-dependent variability in estrogen signaling. Clearly, further investigations are required to clarify the role of palmitoylation, and its perturbation, in estrogen receptor-mediated signaling in cells of the immune system; a better understanding of these mechanisms might elucidate pathways underlying cancer cell-mediated immune modulation during metastasis.

## Palmitoylation and EGF family members

The epidermal growth factor receptor family is composed of four protein-tyrosine kinases (EGFR/ERBB1 and ERBB2–4) that belong to the ErbB lineage of proteins.^84^ Overexpression of EGFR is associated with multiple cancers, tumor progression, and metastasis.^43^ Investigation into the cancer-promoting protein CDCP1 in ovarian cancer has revealed a role for palmitoylation in EGF-stimulated expression of CDCP1.^85^ Under basal conditions CDCP1 is constitutively internalized and degraded. Following EGF exposure, palmitoylation and degradation are blocked and the lifespan of the protein is increased. This study demonstrates a link between palmitoylation events and EGFR signaling, but further work is required to elucidate the exact mechanisms involved.

### EGFR function and FASN-dependent palmitoylation

Fatty acid synthase (FASN) is a major lipogenic enzyme that synthesizes palmitic acid. Overexpression of FASN in normal cells induces an oncogenic phenotype; the transformation involves enhanced lipid synthesis and an increase in phosphorylation and expression of EGFR.^86^ Using prostate and lung cancer cells Bollu *et al.*^43^ showed that EGFR activation involves FASN-dependent palmitoylation; this occurs intracellularly and is required for both EGFR ligand-dependent and ligand-independent activation. Treatment of cells with the FASN inhibitor cerulenin or the palmitoylation inhibitor 2-bromopalmitate (2-BP) reduces EGFR levels at the plasma membrane, and concurrently increases levels at lysosomes. The relationship between FASN and EGFR appears to be bidirectional, and Bollu *et al.* also demonstrated a link between plasma membrane EFGR (pmEGFR) signaling and EGFR present in mitochondria (mtEGFR). Here EGF-activated pmEGFR enhances FASN activity and thus the *de novo* production of palmitate, which subsequently acts as a signal molecule for mtEGFR, inducing palmitoylation-dependent activation of the receptor.^87^ Bollu and colleagues emphasize the implications of their findings in regard to therapeutic approaches that have been developed based on the understanding of ligand-dependent and EGFR kinase activity-dependant mechanisms; awareness of signaling circuits that are independent of ligand and kinase activity provides opportunity to improve drug efficacy and to better understand pathways to resistance.

### Evidence for palmitoylation-dependent regulation of ERBB2

ERBB2 (also referred to as HER2/neu) is expressed at low levels in normal breast epithelial cells^88^ and overexpressed in about 15 to 25% of breast cancers.^89^ ERBB2 was first identified as a tumor antigen in cell lines from rat in which neuro/glioblastoma cancer had been chemically induced.^90^ ERBB2 is unique within the EGF family in that no known soluble ligand has been identified, and it does not contain a key intramolecular ‘tether’ in the extracellular region that autoinhibits other human EGF family members.^91^ As with EGFR, FASN phosphorylation, and subsequent activity have been associated with ERBB2 overexpression,^92,93^ and in one study changes in protein level were not reflected at the messenger RNA levels indicating that FASN-ERBB2 interaction is mediated post-transcriptionally. The Bollu group found that the cysteine residue for EGFR palmitoylation (Cys797) is conserved across EGFR, ERBB2 and ERBB4, all of which exhibit kinase activity, but is substituted by a serine in ERBB3, which lacks kinase activity. Furthermore, mutation of Cys797 in each EGFR family member blocked palmitoylation and ERK signaling in non-cancer cell lines (HEK293T).^43^

Prior to the work by Bollu *et al.*, a crystallographic study hinted at the possibility of palmitoylation-dependent interaction between ERBB2 and non-soluble proteins.^91^ The authors conducted structural comparisons between human ERBB2 and the single EGF receptor in *Drosophila melanogaster* dEGFR. The latter has four ligands, one of which (Spitz) has been shown to require palmitoylation for plasma membrane association, and two others (Gurken and Keren) contain a similar palmitoylation site. The structural similarity of ERBB2 to dEGFR prompted the authors to posit that ERBB2 and dEGFR may both be regulated via palmitoylation-dependent activation involving membrane-bound as opposed to soluble ligands. In human, proteins that support this conjecture include Mucin 4 (MUC4) and the ERBB2-interacting protein ERBB2IP. The latter, also known as Erbin, was first described as a binding partner of ERBB2, and is dependent on palmitoylation for membrane localization. ERBB2IP is implicated in ERBB2-dependent tumor growth and cell migration.^94–96^ MUC4 is a cell-surface glycoprotein that also interacts with ERBB2 and promotes its tyrosine phosphorylation.^97^ At the time of writing, no reports describing palmitoylation of MUC4 have been identified but the modification is reported for other members of the mucin family: Mucin 2 (MUC2) undergoes N-terminal palmitoylation and is regulated by FASN in the colon;^98^ and recycling to the cell surface of Mucin 1 (MUC1), which modulates EGFR activity, requires palmitoylation of both cysteines within a CQC motif at the boundary of the transmembrane and cytoplasmic domains.^52^ Interestingly, both ERBB2IP and MUC4 have been associated with resistance to the ERBB2 antibody trastuzumab.^94,99^

### Important knowledge gaps relating to EGF signaling

Palmitoylation of integrin subunits beta-4 (ITGB4), alpha-3 (ITGA3), and alpha-6 (ITGA6) impacts on the formation of integrin tetraspanin complexes.^100^ ITGA6 and ITGB4 cooperate with EGFR and ERBB2 to facilitate multiple aspects of tumor progression and metastasis.^101^ For example, in a mouse model of ERBB2-mediated breast cancer, ITGB4 forms a complex with ERBB2 and amplifies ERBB2 signaling. Loss of ITGB4 in this model reduced invasive growth and metastasis.^102^ Given recent evidence showing a role for palmitoylation in EGF receptor signaling it will be important to undertake studies to determine how different palmitoylation perturbation pathways impact on EGF receptor-integrin dynamics within tetraspanin complexes. It is envisaged that mutation-based approaches that eliminate the activity of specific palmitoyltransferases and/or target substrates will be useful in this regard.

## Palmitoylation and breast cancer stem cell markers

Inflammatory conditions within the microenvironment and aberrant epithelial–mesenchymal transition (EMT) are thought to confer stem cell properties to carcinoma cells.^4^ These transformed cells have enhanced capacity for migration and invasion and are commonly referred to as cancer stem cells (CSCs). CSCs are characterized by the expression of specific surface markers including EpCAM^+^ CD24^−/low^/CD44^+/high^ and ALDH^+^. The latter cells are epithelial-like and associate with a self-renewal phenotype, whereas EpCAM^+^ CD24^−^/CD44^+^ are associated with the metastatic and aggressive mesenchymal phenotype.^103^ CD44 is a large transmembrane glycoprotein that acts as a receptor for hyaluronic acid. Transcripts of this gene undergo complex alternative splicing to produce functionally distinct isoforms, and several post-translational modifications including palmitoylation have been reported.^104^ Specifically, the mutation of either of two highly conserved cysteine residues reduces the incorporation of palmitate, prevents association with CD44-lipid rafts, and inhibits the internalization of hyaluronan.^105^ Palmitoylation is thus a crucial determinant of CD44 turnover at the cell surface, and of hyaluronic acid endocytosis. In a rat model of colorectal cancer, a specific isoform of CD44, CD44v6, forms a complex with Claudin-7, a tetraspanin (TSPAN8/ CO-029) and EpCam. It has been suggested that this complex, rather than the individual proteins, supports tumor progression and metastasis.^106^

### Gaps in current knowledge

EpCam co-localizes with CD44, Claudin-7, and TSPAN8 in tetraspanin-enriched membrane microdomains. The role of palmitoylation in the formation of these membrane complexes is only partially understood. The stabilization and interaction among tetraspanins is reported to be palmitoylation-dependent, but a role specific to TSPAN8 function is yet to be investigated.^107^ Palmitoylation of Claudin-7 is essential for association with, and recruitment of, EpCAM to glycolipid-enriched membrane domains in HEK cells.^108^ At the time of writing, no reports describing palmitoylation of EpCAM itself have been identified, but an extracellular domain within the N-terminal of the protein contains tandem EGF-like repeats.^109^ Mutational approaches show that these repeat domains are essential for accumulation of EpCAM molecules at cell–cell boundaries, lateral interaction between molecules, and anchorage to F-actin, all of which could potentially be palmitoylation dependent.^109^

## Palmitoylation and immune modulation

To maintain immune homeostasis, pro-, and anti-inflammatory pathways act concurrently as opposing forces. Perturbation of the dynamic balance between them leads to immune-mediated disease including cancer progression and metastasis.^5^ In the latter, perturbation of cell population dynamics is an emerging theme. Essentially, the ratios between different phenotypes of the same cell-type become skewed, causing a pathological tip in the equilibrium toward either pro- or anti-inflammatory conditions. A study using a murine model of spontaneous breast cancer provides a case in point.^110^ This study describes a domino effect in which tumor-induced inflammation triggers signaling that leads to loss of the cytotoxic CD8 T lymphocytes that would normally supress neoplasia and metastasis. Cell-depletion approaches demonstrated that neutrophils were essential to this domino effect. The proportions of CD8+ T cells in the lungs of control and neutrophil-depleted tumor-bearing mice were similar, but importantly the number of (metastasis-preventing) effector T cells was enhanced in the latter. Although this example is specific to one mouse model, the concepts described, in particular changes in ratios between phenotypes of the same cell population, are increasingly reported in the breast cancer metastasis literature. In addition to the many subtypes of T cells, neutrophils, and macrophages exhibit both pro- (N1, M1) and anti-inflammatory (N2, M2) phenotypes, the ratio of which can become skewed within the cancer microenvironment. This domino affect involves upregulation of *NOS2* by >150-fold.^110^ *NOS2* encodes inducible nitric oxide synthase (iNOS), and palmitoylation of this protein is required for its proper sorting, localization to the endoplasmic reticulum, and nitric oxide synthetic activity.^111^ It will be important to determine whether, and how, aberrant palmitoylation contributes to the skewing of cell phenotypes as described during the domino effect.

The palmitome of primary and Jurkat T cells was recently evaluated, revealing a pool of 120 palmitoylated proteins common to both cell types. This pool contained well-known palmitoylated proteins plus 92 previously unreported as palmitoylated *in vivo.*^81^ Known palmitoylated proteins include the breast cancer-related proteins ERBB2IP, FASN, CD44, and products of 52 genes implicated in breast cancer metastasis to bone, lung, or brain (Table 1). The T-cell study also showed that some PAT enzymes are themselves palmitoylated, indicating a feedback mechanism, and differential palmitoylation of T-cell surface antigen (mono- versus dual-lipidation) was observed that likely affords greater functional plasticity. The T-cell receptor (TCR) is purported to be one of the best examples of the importance of protein palmitoylation for cell signaling.^112^ Although the TCR subunits do not undergo palmitoylation, the functions of co-receptors CD4 and CD8 and associated signaling molecules including SRC family kinases LCK and FYN and linker for activation of T cells are palmitoylation dependent. Altogether, these lines of evidence provide insight into how global changes in palmitoylation dynamics might be a common feature linking dysregulation of classical breast cancer biomarkers, stem cell surface markers and immune cell function.

### Knowledge gaps in relation to chemokine signaling

Chemokines have a critical role in immune cell function including, for example, maintaining the balance between neutrophil release and retention.^113^ By analogy with palmitoylation-dependent function in immune cells, potential involvement of aberrant palmitoylation in breast cancer warrants clarification. In addition to the palmitoylation-dependent function of the CCR5 receptor (above), in T lymphocytes the function of another breast cancer-associated ligand, CXCL12, depends on dual acylation with myristic and palmitic moieties of a specific isoform of the SRC-family kinase member HCK.^114^ In another example, the ligand CCL18 has been identified as the main driving factor in a deleterious positive-feedback loop, in which CSCs activate macrophages with an anti-inflammatory M2-like phenotype. Reciprocally, the latter express EMT-inducing molecules that sustain the CSC population.^115^

Chemokine receptors are G-protein-coupled receptors (GPCR), most of which undergo palmitoylation at one, two, or three residues in the carboxyl-terminal cytoplasmic tail. All aspects of GPCR function including coupling to G proteins and strength of signaling have been associated with palmitoylation; however, effects vary across receptor types, and in many cases are yet to be fully characterized.^78^ Similarly, the regulation of palmitoylation depends on the type of receptor and involves a variety of mechanisms including activation by an agonist, changes in nitric oxide levels and/or lipid raft conditions (e.g., cholesterol depletion). Important questions remain to be addressed including why the attachment of palmitate to the same domain can subserve the many different functional roles of GPCR proteins.^78^ It is likely that different combinations of PTMs acting concurrently within a protein, and/or PTM events occurring simultaneously across multiple proteins, coenact specific biological effects. Palmitoylation-dependent regulation of chemokines is less understood, but likely mirrors the complexity and variation described for the broader family of GPCRs.

## Interplay among PTMs including retinoylation

Palmitoylation events are part of a complex interplay involving several types of PTMs. Multiple lipid modifications can co-occur (e.g., palmitoylation and prenylation and/or myristoylation)^116^ and can act concurrently, sequentially, or antagonistically with other protein modifications including phosphorylation and ubiquitination.^117,118^ It has been shown, for instance, that *O*-linked acylation of serine is required for subsequent palmitoylation of cysteine in mouse Wnt proteins,^119^ and that palmitoylation enhances the interferon-induced transmembrane protein IFITM3, while ubiquitination decreases activity.^118^ Overall, the interdependency of PTMs occurring within a single protein is only just beginning to be described. Of particular interest to breast cancer biology is retinoylation (acylation by retinoic acid).

Takahashi and Breitman^120^ first presented evidence for covalent attachment of retinoic acid (RA) to protein in 1989. They subsequently showed that some proteins from MCF-7 cells undergo retinoylation and are also targets for E2 ligands.^121^ Treatment with tunicamycin, an inhibitor of both protein *N*-glycosylation and palmitoylation, decreased palmitoylation while enhancing retinoylation of proteins, indicating a potential opposing relationship between these modifications.^122^ XPO1 is an example of a protein potentially regulated by both palmitoylation and retinoylation, with functional relevance to breast cancer.

The karyopherins (e.g., KPNA1 and KNPB1) and XPO1 are nuclear transporters that shuttle proteins between the nucleus and the cytoplasm.^123^ Analysis of cultured rat embryonic neurons identified XPO1 as a potential candidate for palmitoylation.^124^ This protein is also a member of the palmitome of human primary and Jurkat T cells.^81^ Curcumin, which has been shown to block the auto-acylation step of the ZDHHC3 PAT,^125^ was found to target XPO1 and specifically interact with cysteine (Cys528).^126^ XPO1 undergoes retinoylation at the same residue that is targeted by curcumin (Cys528).^79^ Altogether, these lines of evidence suggest that XPO1 undergoes both palmitoylation and retinoylation.

Most transcription factors implicated in EMT contain conserved signal sequences that are recognized by karyopherins.^123^ Inhibition of XPO1 was found to reverse EMT in snail-transduced primary human mammary epithelial cells, and to completely eliminate tumors in a xenograft mouse model.^123^ XPO1-dependent protein shuttling is also important within the immune context. For example, the cellular release of proinflammatory TNF-alpha from stimulated neutrophils is XPO1-dependent.^127^ It would be interesting to determine whether retinoylation and palmitoylation differentially impact on XPO1 function, and if so whether perturbation of this dynamic contributes to breast cancer metastasis.

Retinoylation is inhibited or enhanced by palmitic and arachidonic acids, respectively. These fatty acids are products of phospholipase 2 (PLA2), and PLA2 activity is part of the functional repertoire of the tumor suppressor RARRES3. This gene was initially named retinoid acid receptor responder (tazarotene induced) 3 (*RARRES3*) due to its upregulation in human keratinocytes that had been exposed to the synthetic retinoid tazarotene.^128^ It has been extensively renamed and is most recently described as member 4 of the H-RAS-like suppressor (HRASLS) subfamily (HRASLS4). This family consists of five enzymes that display phospholipase A1/2 and *N*- and *O*-acyltransferase activities.^27^ The tumor suppressor effects of RARRES3 have been attributed, by two investigative studies, to its activity as a phospholipase^129^ and, as mentioned earlier in this review, as an APT (due to its capacity to reduce palmitoylation levels of Wnt proteins).^26^ In the former study, phospholipase activity was associated with cellular changes in arachidonic acid. It is interesting to speculate that increased arachidonic acid, and possibly subsequent increase in cellular retinoylation, may have indirectly contributed to the reduced Wnt protein palmitoylation observed in the latter study, as opposed to direct ATP-enacted depalmitoylation.

## Conclusions

Aberrant protein palmitoylation during cancer represents an area of increasing interest. Research efforts have focussed on palmitoylation, but other forms of *S*-acylation likely have important roles in protein regulation. The lines of evidence presented herein indicate that aberrant palmitoylation may be a factor common to the dysregulation of primary breast cancer markers, chemokines and other molecules involved in tumor-induced immunosuppression. These insights raise interesting avenues for future research. For instance, viewing the relationship between estrogen and retinoid-related signaling through the lens of palmitoylation and retinoylation dynamics might improve our understanding of estrogen/retinoic acid antagonism and/or agonism described in breast cancer.^130,131^ Research into the relationship between estrogen and retinoid pathways has primarily focussed on classical retinoid acid receptor signaling, whereas non-classical pathways described in retinoylation,^79^ and in regard to the role of aldehyde dehydrogenases and cancer stem cells^132^ are perhaps more worthy of investigation within the context of post-translational protein regulation. Similarly, the interplay and possible interdependency among PTMs is only just beginning to be understood; perturbations arising here may represent causal mechanisms that drive ‘symptoms’ presenting at the transcriptional level, elucidating potent therapeutic targets. The development of highly specific palmitoylation inhibitors will likely prove important, but it may be that dysfunction is a consequence of substrate competition or imbalance in the cellular concentrations of palmitate and other moieties (e.g., arachidonic acid) as opposed to aberrant function of PATs and APTs.

Palmitoylation-specific investigations are required to help address the many fundamental questions that remain unanswered. It would be useful to evaluate the palmitome in CSCs extracted from patients with different breast cancer subtypes and/or stages of disease; compare palmitoylation events between tumor and control samples; and assess the influence of estrogen, retinoid, and EGF signaling pathways on known palmitoylation-dependent proteins that regulate immune cells within the tumor microenvironment.

We believe that RARRES3 is an ideal candidate for further enquiry at the enzymatic level to clarify, for instance, the functional consequences of multiple lipid-related modifications conferred concurrently or in rapid succession by a single enzyme. It is also possible that this enzyme has capacity to mediate retinoylation, for which enzymatic modifiers have yet to be described. We expect that a more-comprehensive understanding of palmitoylation will lead to novel therapeutic strategies for prevention and cure of breast cancer metastasis.

## Figures and Tables

**Table 1:** Genes encoding T-cell palmitoylated proteins that have also been implicated in tissue-specific breast cancer metastasis

*Reference*	*Symbol*	*Gene name*
Bos *et al.*^36^	*ATP2B4*	ATPase, Ca++ transporting, plasma membrane 4
	*CD99*	CD99 molecule
	*GSTP1*	Glutathione *S*-transferase Pi 1
	*IL32*	Interleukin 32
	*LSS*	Lanosterol synthase (2,3-oxidosqualene-lanosterol cyclase)
Kang *et al.*^37^	*CXCR4*	Chemokine (C–X–C motif) receptor 4
	*FYN*	FYN proto-oncogene, Src family tyrosine kinase
	*MCAM*	Melanoma cell adhesion molecule
	*PTK7*	Protein tyrosine kinase 7 (inactive)
	*SCAMP4*	Secretory carrier membrane protein 4
Minn *et al.*^134^	*ATP11A*	ATPase, Class VI, Type 11A
	*DAAM1*	Dishevelled associated activator of morphogenesis 1
	*STOM*	Stomatin
Sevenich *et al.*^135^	*ADAM17*	ADAM metallopeptidase domain 17
	*ANXA6*	Annexin A6
	*ARF1*	ADP-ribosylation factor 1
	*ARF3*	ADP-ribosylation factor 3
	*CANX*	Calnexin
	*CAPN5*	Calpain 5
	*CBX3*	Chromobox homolog 3
	*DAD1*	Defender against cell death 1
	*EEF2*	Eukaryotic translation elongation factor 2
	*EIF3M*	Eukaryotic translation initiation factor 3, subunit M
	*GDI2*	GDP dissociation inhibitor 2
	*HNRNPA1*	Heterogeneous nuclear ribonucleoprotein A1
	*HNRNPK*	Heterogeneous nuclear ribonucleoprotein K
	*HP*	Haptoglobin
	*HSP90AB1*	Heat shock protein 90 kDa alpha (Cytosolic), class B member 1
	*ILF2*	Interleukin enhancer binding factor 2
	*KARS*	Kirsten rat sarcoma viral oncogene homolog
	*NONO*	Non-POU domain containing, octamer-binding
	*NPM1*	Nucleophosmin (nucleolar phosphoprotein B23, numatrin)
	*PSMA6*	Proteasome subunit alpha 6
	*PSMC1*	Proteasome 26S subunit, ATPase 1
	*PSMC2*	Proteasome 26S subunit, ATPase 2
	*PSMC3*	Proteasome 26S subunit, ATPase 3
	*PSMC4*	Proteasome 26S subunit, ATPase 4
	*PSMC5*	Proteasome 26S subunit, ATPase 5
	*PSMC6*	Proteasome 26S subunit, ATPase 6
	*PSMD1*	Proteasome 26S subunit, Non-ATPase 1
	*PSMD13*	Proteasome 26S subunit, Non-ATPase 13
	*PSMD2*	Proteasome 26S subunit, Non-ATPase 2
	*PSMF1*	Proteasome inhibitor subunit 1
	*REEP5*	Receptor accessory protein 5
	*RPL10A*	Ribosomal protein L10a
	*RPL27*	Ribosomal protein L27
	*RPL6*	Ribosomal protein L6
	*RPL9*	Ribosomal protein L9
	*RPS5*	Ribosomal protein S5
	*RPS6*	Ribosomal protein S6
	*SLC25A3*	Solute carrier family 25 (mitochondrial carrier; phosphate carrier), member 3
	*SNRNP200*	Small nuclear ribonucleoprotein 200 kDa (U5)
